# Persistent cornual pregnancy mimicking uterine arteriovenous malformation: a case report

**DOI:** 10.1186/s12905-023-02450-9

**Published:** 2023-06-16

**Authors:** Zhuolin Xie, Yang Wang, Rong Chen

**Affiliations:** Department of Obstetrics and Gynecology, Peking Union Medical College Hospital, National Clinical Research Center for Obstetric & Gynecologic Diseases, Chinese Academy of Medical Sciences & Peking Union Medical College, Beijing, People’s Republic of China

**Keywords:** Uterine arteriovenous malformation, Persistent ectopic pregnancy, Pregnancy residue

## Abstract

**Background:**

Uterine arteriovenous malformation(AVM) refers to the abnormal direct traffic between uterine arteries and veins, which can be characterized by the imaging examination, showing increased uterine vascularity and arteriovenous shunting. However, similar imaging manifestations can also be seen in a variety of conditions including retained production of conception, gestational trophoblastic disease, placental polyp, and vascular neoplasm.

**Case presentation:**

Here we present a case of a 42-year-old woman who was suspected of suffering uterine AVM indicated by Doppler sonography and magnetic resonance imaging but was finally diagnosed with a persistent ectopic pregnancy located on the right uterine corner by pathology after laparoscopy. She recovered well after surgery.

**Conclusion:**

Uterine AVM is a rare and serious condition. In general, it presents special radiological manifestations. However, when complicated with other diseases it can also be distorting. Standardized diagnosis and management are important.

## Background

Uterine arteriovenous malformation (AVM) is one of the rare uterine vascular formations which can be acquired or congenital [1]. Acquired AVM is often secondary to previous pelvic surgery, infection, cesarean section, gestational trophoblastic neoplasia, or endometrial neoplasia [2]. It is characterized by a single direct arteriovenous communication between the branches of the uterine artery and the myometrial venous plexus [3]. AVM can cause abnormal vaginal bleeding and pelvic pain, or sometimes it can be asymptomatic [1]. The discovery of AVM requires imaging examinations. Under Doppler ultrasonography (US), it typically shows a tubular and tortuous hypoechoic structure with mixed signals at color-Doppler coming from arterial and venous flows into the myometrium, while under magnetic resonance imaging (MRI) the involved portion of the uterus and the vascular supply of the AVM can be identified [3].

However, AVM’s clinical presentation and imaging features can be mimicked, especially similar to pregnancy residue [4] or other persistent existence of pregnancy production. Thus, attention should be paid to the identification of the pregnancy residue and post-abortion AVM with similar imaging findings. Here we present a case of a 42 years-old woman who was once considered to suffer from AVM but after surgery, we found out her final diagnosis was a persistent ectopic pregnancy, which may or may not be complicated with uterine AVM.

## Case Presentation

We present a case of a 42-year-old female gravida 2, para 0 (G2P0) who was admitted to our hospital with a suspected uterine arteriovenous malformation indicated by US and MRI.

The patient was implanted with an embryo by assisted reproduction technology on March 1, 2022. At the end of March, presenting mild vaginal bleeding, she did US examinations twice in two days, one showed the gestational sac was located in the right uterine horn, considered a cornual pregnancy, while the latter one indicated that the embryo was only near the right uterine horn but still inside the uterine cavity, so she didn’t do the further examination or treatment. The inconsistent ultrasound images perhaps were influenced by subjective factors of different diagnosticians. On April 4, 2022, she was diagnosed with embryo demise and had a drug-induced abortion, after which the US examination was performed immediately and showed no residue, thus the curettage wasn’t done. The patient underwent four times failed embryo transfers in the past two years and went through a hysteroscopic surgery due to repeated transplant failure, which found and resected intrauterine adhesion. There was nothing else special in her other past medical histories.

**Fig. 1:**
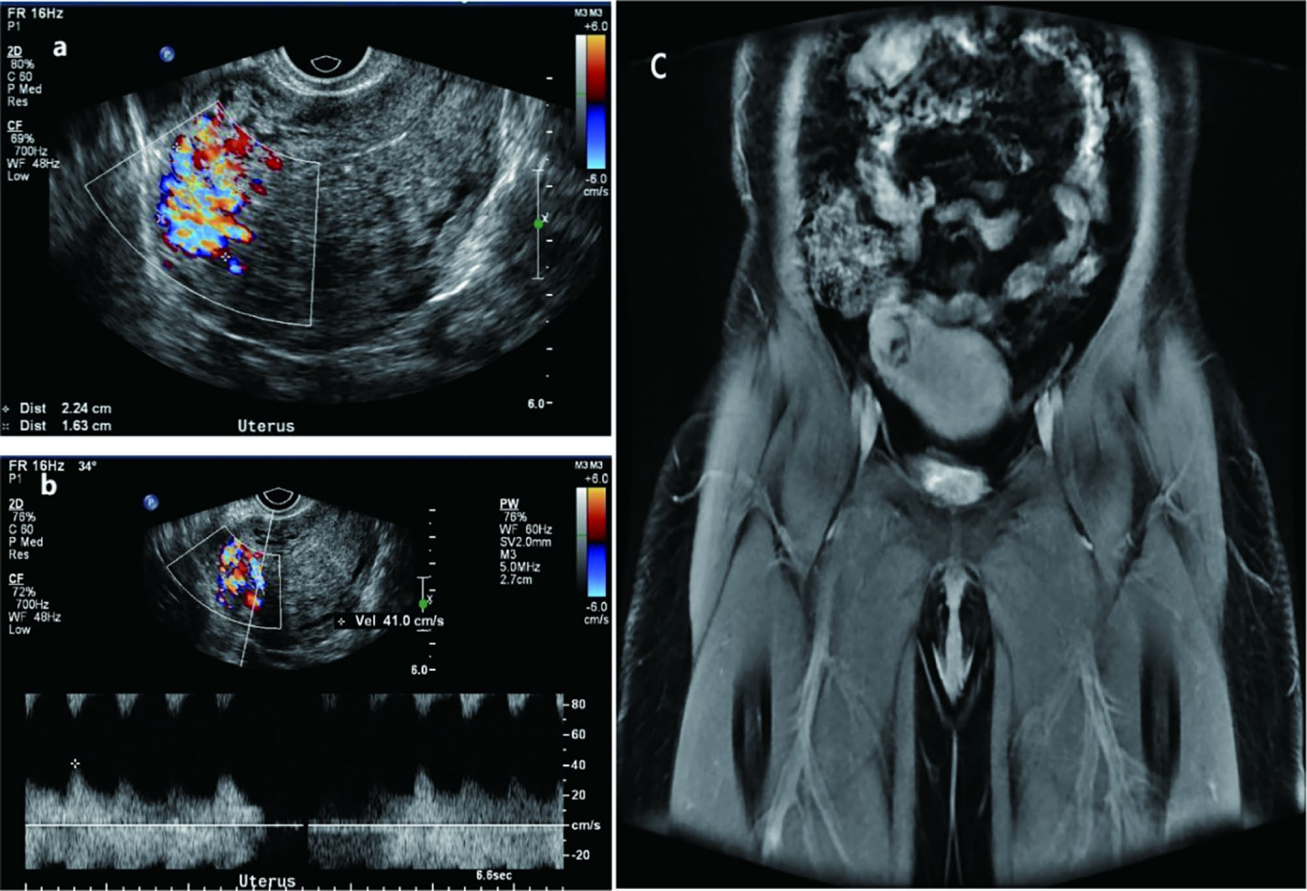
(**a**) An area with abnormally rich blood flow signals in the right region of the myometrium was about 2.2 × 1.6-cm. Another mixed echo area, 3.4 × 3.4 × 3.3-cm, was in the right uterine horn. (**b**) Spectral waveform demonstrated high velocity (50 cm/s), continuous high flow through diastole and systole, and low-resistance waveform. (**c**) Multiple tortuous flow voids could be seen in an obviously bulging area on the right side of the uterus

She did the transvaginal sonography because of mild cramping in the right lower abdomen in early July and found a suspected uterine arteriovenous malformation. Doppler US revealed an area that was 2.2 cm×1.6 cm in the right region of the myometrium with abnormally rich blood flow signals, as well as another mixed echo area, 3.4 cm×3.4 cm×3.3 cm, in the right uterine horn, which showed tortuous dilated veins and a high-velocity low-resistance signal pattern of blood flow with the arterial waveform on spectral analysis, with a peak systolic velocity of 50 cm/s (Fig. 1a and b). The patient underwent contrast-enhanced magnetic resonance imaging (MRI) of the pelvis. Multiple tortuous flow voids in an obviously bulging area on the right side of the uterus (Fig. 1c).

**Fig. 2:**
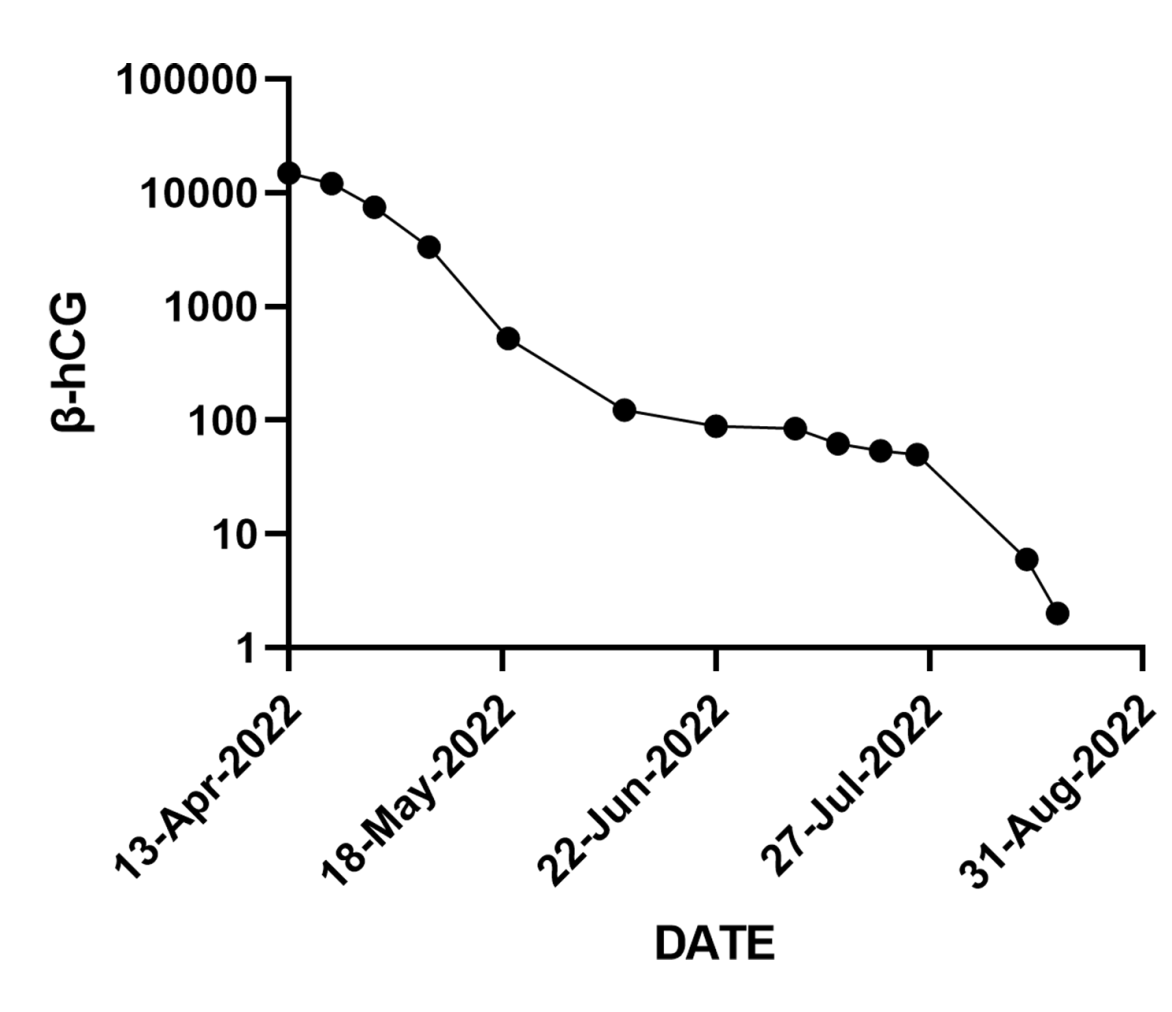
The change of β-hGC(β-human chorionic gonadotropin)

After the abortion, she failed to restore a regular period, with only a small amount of vaginal bleeding at the beginning of June, July, and August which may be menstruation. Her β-human chorionic gonadotropin (β-hCG) slowly decreased but was constantly above normal (Fig. 2).

She was suspected diagnosed with uterine AVM when admitted to our hospital on 2022 Aug 9. Because the patient’s β-hCG hadn’t dropped to normal, the possibility of complications with residual pregnancy material or gestational trophoblastic disease couldn’t be ruled out. Doppler US was repeated after hospitalization on August 9, which showed abnormal blood flow signals in the right-sided region of the fundus uterine muscle, which was 2.9 × 2.3 cm, PSV = 55 cm/s. A mix echo area, 4.4 × 4.1 × 3.3 cm, was seen on the right uterine corner. Dilates venous vessels could be seen in this area with pulsatile blood flow spectrum, PSV:51 cm/s, and RI:0.30.

**Fig. 3:**
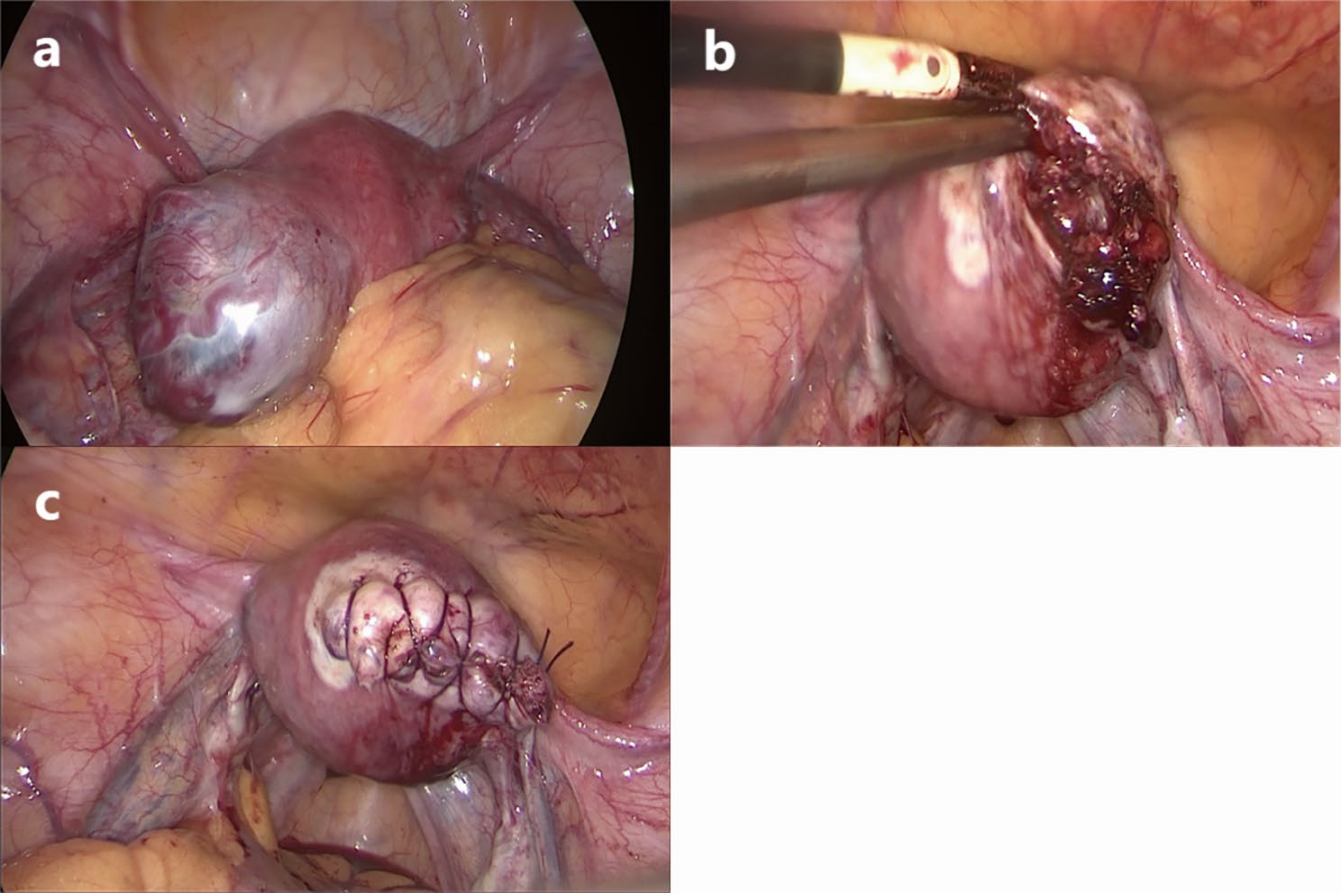
(**a**) The lesion was located on the right side of the uterus. (**b**) Stale dull red organize was seen inside the bulge. (**c**) The incision was closed in two layers using a continguous absorbable suture

After professional groups’ discussion and adequate preoperative preparation, laparoscopy and hysteroscopy were performed on August 10. Being fully aware of the operative bleeding risk, we booked 8 units of red blood cells (RBC) and 800ml plasma in our hospital blood bank. Laparoscopy was performed using 4 ports: 1 placed in the sub umbilical area (10 mm), 2 in the left lower abdomen (5 mm), and 1 in the right lower abdomen (10 mm). Under laparoscopy, the right corner of the uterine was externally bulging with dilated uterine vessels on its root and surface(Figure 3a). Before cutting open, 2 units of pituitrin were dispensed into 40ml normal saline then local injection was made at the lesion site. Bipolar electrocauterization was gently used on the surface of the bulge to minimize bleeding. Bipolar incised the bulge and exposed stale dull red organize. Through sharp and blunt dissection, the abnormal tissue inside the bulge was completely removed and did not pierce the uterine cavity (Fig. 3b). The incision was closed in two layers using a continuous absorbable suture (Fig. 3c). All specimens were sent for pathological examination.

**Fig. 4:**
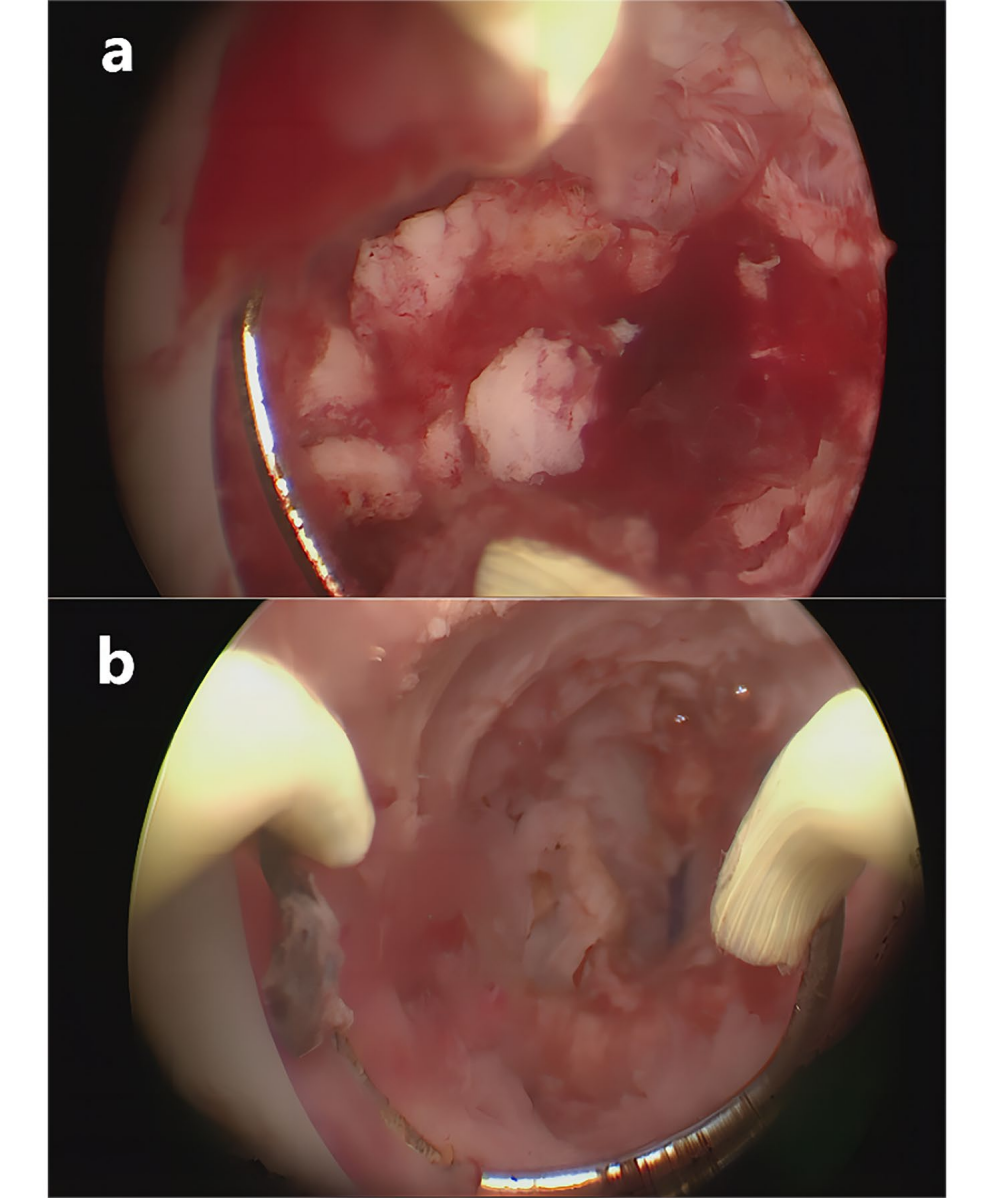
(**a**) The muscular adhesion band was in the lower-middle segment of the uterine. (**b**) uterine cavity

Hysteroscopy was performed under laparoscopy for visual monitoring. A muscular adhesion band could be seen in the lower-middle segment of the uterine. After removed the band, the uterine cavity was exposed (Fig. 4), which was morphologically irregular, indicating a severe intrauterine adhesion. The Bilateral tubal opening was invisible.

The patient recovered well after surgery and was discharged on postoperative day 2. The repeat β-hCG level was 6 IU/L on postoperative day 2 and turned negative on outpatient follow-up one week later after surgery. The regular menstruations returned 1 month postoperatively. However, she felt the bleed length was shortens from 5 days to 3 days, while the amount of bleed was less. The post-operative pathology was stale gestational tissue with a little smooth muscle tissure, which indicated the diagnosis of this patient should be a persistent ectopic pregnancy, which was located on the right uterine corn. However, acquired uterine AVM couldn’t be ruled out.

## Discussion

Uterine arteriovenous malformations are rare and vary with presentations, they can represent a life-threatening condition because of severe hemorrhage [5], or sometimes can also remain asymptomatic and diagnosed incidentally [3], just like the patient presented in our case. The acquired uterine AVM can be secondary to prior uterine surgery, spontaneous abortion, direct uterine trauma, endometrial cancer, or gestational trophoblastic disease [6]. Sometimes it can also appear in cases of abortion and natural pregnancies [7]. Especially, after necrosis of chorionic villi in retained products of conception, arteriovenous communications can develop [4], thus explaining the findings in our case. Due to its rarity and diagnostic challenges, our clinical knowledge of AVMs is limited [8]. The diagnosis dilemma of this case is the acquired uterine AVM complicated by persistent uterine corner pregnancy.

Increased uterine vascularity and arteriovenous shunting showed on Doppler ultrasound, as well as serpiginous vascular structures arerter shown on enhanced MRI, are imaging features that may suggest AVM but are not pathognomonic since they can also be seen in retained products of conception, gestational trophoblastic disease, placental polyp, and vascular endometrial neoplasm. MRI angiography is the gold standard in the diagnosis of AVM. In our case, the patient didn’t do the angiography due to its invasion.

AVM management varies significantly due to the broad spectrum of clinical presentations and the differentia of severity. Patients’ fertility willingness should also be considered. So far, there is no clear consensus regarding AVM management [9]. For conservative management, medications have been tried, including methotrexate, misoprostol, methylergometrine maleate, and gonadotropin releasing hormone (GnRH) agonists [10]. Embolotherapy is considered an alternative to surgical intervention [3]. When conservative treatments fail, for women who have already finished their birth plan, a hysterectomy remains the common recommendation [11]. Fertility-preserving surgeries such as laparoscopy and hysteroscopy often are reported as case reports [12, 13]. In our case, medications and surgeries were tried. Embolotherapy wasn’t performed due to the mild clinical symptoms of this patient, which only presented as small vaginal bleeding.

The coexistence of AVM with persistent existence products of conception is a rare condition. In light of its uniqueness, some investigators recommend differentiating the acquired uterine arteriovenous malformations associated with retained products of conception (UAVM-RPOC) from other types of uterine AVMs [10]. We think that this concept has great reference value for our case.

In our case, the patient was a persistent ectopic pregnancy in which the embryo was located on the right corner of the uterine and the lesion obviously bulged outward. Ectopic pregnancies account for 2% of all pregnancies and the majority are tubal pregnancies, while non-tubal ectopic pregnancies (NT-EPs) only account for 5-8.3% of all ectopic pregnancies but can be potentially life-threatening conditions [14]. The gestational sac could be implanted on the cervix, ovary, uterine corner, abdomen, interstitial portion of the fallopian tube, cesarean scars, and so on. Meanwhile, our patient was considered as a persistent ectopic pregnancy due to a failed drug-induced abortion and a remaining elevated β-hCG for 4 months after abortion. The most possible type of ectopic pregnancy in our case was right uterine corn pregnancy, as her US examination once showed the gestational sac was located in the right uterine horn. When the gestational sac expands and grows outside the uterine cavity, it will cause the uterine horn to expand and protrude, while the muscular tissue of the uterine horn gradually thins out, just as we saw under laparoscopy. However, some researchers prefer to define cornual pregnancy as “a conception that develops in the rudimentary horn of a uterus with a mullerian anomaly”, while in general practice, radiologists, ultrasonographers, and practitioners still used to use the term cornual pregnancy refers to the gestations that occur near the uterine cornua, where is the junction of oviduct and uterus, no matter in the normal or anomalous uterus [15]. The main differential diagnosis was interstitial pregnancy and subserosal pregnancy. Interstitial pregnancy defines as a gestation that implants within the proximal tubal segment that lies within the muscular uterine wall [15]. Subserosal pregnancy is a newly defined intramural pregnancy, which means the gestational sac was surrounded only by the serosa of the uterus [16]. All three types of ectopic pregnancy mentioned above could appear as a lesion that obviously protrudes at the uterine corner, however, considering the ultrasound results before the abortion, we preferred to believe her ectopic pregnancy was a right uterine corn pregnancy. Meanwhile, the postoperative pathological diagnosis showed smooth muscle tissue, which indicated that it was less likely to be a subserosal pregnancy, as it was characterized by the absence of myometrium surrounding the gestational sac [16]. Nevertheless, it should be noticed that in this case, the persistent ectopic pregnancy was a retrospective diagnosis. Even though we considered the cornual pregnancy as the most likely diagnosis, other types of ectopic pregnancy couldn’t be completely ruled out, which wouldn’t affect the final management of our patients. As one of the pregnancy-related disorders, we believe persistent ectopic pregnancy associated with UAVM owns a similar nature and characteristics as UAVM-RPOC.

The treatment of UAVM-RPOC is based on bleeding risk and clinical presentation rather than imaging features [10]. One retrospective study focused on UAVM-RPOC using conservative management, uterine artery embolotherapy, dilatation and curettage (D&C) and operative hysteroscopy found that all the treatments were successful and all patients were followed for 1 year, and no recurrences were observed [17]. However, laparoscopy didn’t be used in this research for their patients were residual pregnancies secondary to intrauterine pregnancies. In our case, due to the lesion was clearly bulged out, we choose laparoscopy and hysteroscopy as our treatments and mostly removed the lesion under laparoscopy.

In conclusion, uterine AVM is a rare condition, especially when complicated by retained products of conception or the persistent existence of ectopic pregnancy. Imaging findings and radiological outcomes can be distorting, so careful and specific diagnosis and management are important. Special consideration is required for a patient with fertility goals.

## Data Availability

All data for the case reports are available in this manuscript.

## Abbreviations

AVM: arteriovenous malformation
US: ultrasonography
MRI: magnetic resonance imaging
β-hCG: β-human chorionic gonadotropin
RBC: red blood cell
GnRH: gonadotropin releasing hormone
UAVM-RPOC: uterine arteriovenous malformations associated with retained products of conception
NT-EPs: non-tubal ectopic pregnancies
D&C: dilatation and curettage
